# Beyond the Battlefield: Geospatial Insights of Health Vulnerability in Ukraine During the Russian-Ukrainian War

**DOI:** 10.21203/rs.3.rs-7376114/v1

**Published:** 2025-08-18

**Authors:** Ubydul Haque

## Abstract

Although the devastation of war is well recognized, no previous study has systematically integrated the interplay of conflict intensity, access to humanitarian aid, environmental conditions, and infrastructure to understand how these factors collectively shape health vulnerability across war-affected regions. Using spatial and suitability modeling, we assessed multidimensional vulnerabilities in Ukraine during the Russian invasion, including mental health risks, environmental stressors, and infrastructure disruptions. We developed a multi-source conflict-related health impact database (February 2022–December 2023). Sleep, mental health, and casualty data were collected through national online surveys (n > 2,312). Data was cleaned, geolocated across 461 cities, and analyzed using Inverse Distance Weighting interpolation. Logistic and spatial regression were used to assess relationships between conflict exposure, living conditions, mental health, sleep deprivation, and cold injury risks. A composite vulnerability index was created using weighted Principal Component Analysis-based methods. Regions with intense conflict, poor housing, frequent power outages, food shortages, and limited access to healthcare and aid faced the highest vulnerability. Cold and damp conditions, housing damage, and resource scarcity exacerbated household living conditions, especially in eastern and northern Ukraine. Not all high-conflict areas had poor mental health outcomes; cold, damp, crowded housing, food insecurity, and power outages were equally critical drivers. Mental health issues (PTSD, depression, and insomnia) were highest in regions with harsh winters, poor infrastructure, and limited aid. War-related health issues extend beyond direct conflict exposure and involve the interplay of conflict, environmental stressors, and infrastructure damage in shaping casualties, sleep, and mental health outcomes.

## Introduction

Between 1999–2023, an estimated 4.5 to 4.7 million people have died in conflict areas around the world^1^. In addition to the conflict-related deaths, war hampers social and economic development, devastates health systems, and disrupts community life^2^. Since its start in 2022, the conflict in Ukraine has severely damaged infrastructure, disrupting power, water, and fuel supplies^3^. Damage to critical civilian energy infrastructure in the country caused winter blackouts and environmental stressors (i.e., cold, damp housing, and frequent power outages), increasing the risk of cold-related injuries and exacerbating humanitarian needs, particularly among vulnerable sub-populations. Compounding the crisis, the 2023 Kakhovka Dam destruction caused severe flooding, displacing thousands more Ukrainians and worsening vulnerability^4^.

It has been reported that war refugees face increased vulnerability to mental health disorders due to compounded trauma from war and environmental hardships^5,6^. Understanding how war stress, environment, and social factors affect mental health in conflict zones is vital. Integrating multi-level data on environmental and social factors into risk assessments may help to identify sub-populations at greatest risk of mental health vulnerabilities, and in so doing, it may help shape policies, allocate resources, and create sustainable, impactful interventions^7^. However, vulnerability assessments in conflict zones like Ukraine are hindered by security risks, accessibility issues, and evolving conflicts^8^. Despite those challenges, mapping multidimensional vulnerabilities and underlying community factors is vital for practical risk assessment and planning in conflict-affected, resource-limited settings. This approach supports the development of adaptable strategies and enables more effective, targeted interventions. Moreover, humanitarian planning can become more adaptive and resilient by recognizing these interconnected vulnerabilities^9^. To that end, a composite vulnerability index integrating data on war, environmental, and mental health factors may be valuable. Existing data on mental health, sleep, and conflict severity in Ukraine often lacks regional detail, and the limited use of spatial analyses in Ukraine has hindered support for localized needs. Higher resolution, evidence-based studies are urgently needed to inform effective, targeted interventions in high-risk areas.

Here we investigate three primary questions. First, how do environmental factors - such as cold, damp housing and power outages - affect mental health outcomes in conflict-affected populations? We hypothesize that inadequate housing and infrastructure, particularly cold and damp living conditions, significantly worsen mental health outcomes in conflict zones. Second, do mental health vulnerabilities vary spatially across the conflict region, and how do local environmental conditions and conflict intensity shape these vulnerabilities? We hypothesize that areas experiencing high conflict intensity and environmental stressors will show increased mental health vulnerabilities, with these patterns varying spatially and requiring region-specific interventions. Third, how does access to aid, infrastructure, and essential resources influence mental health in high-conflict areas? We hypothesize that limited access to aid, financial support, and basic services exacerbates mental health challenges, especially in regions frequently subjected to attacks and extensive housing damage. This study fills a critical gap by revealing that areas of highest conflict are not always those with the worst health outcomes, highlighting the need for nuanced, location-specific policy strategies to prioritize aid, mental health support, and infrastructure development.

## Methodology

### Data Collection

Building on a previously described database^10^, we compiled data from online and print news sources in English and Ukrainian, including Al Jazeera, BBC, CNN, Kyiv Independent, Rubryka, Reuters, Institute for the Study of War, and UNICEF through December 2023. Reports covered healthcare facilities, ambulance and pharmacy attacks, civilian deaths and injuries, and damage to civilian infrastructure. Attacks on military targets without civilian casualties were excluded. To avoid duplication, a centralized database tracked hospital destruction, infrastructure damage, shelling, and lethal events, with details on dates, locations, and casualties. Additional data on attacks on hospitals, maternity wards, nuclear plants, homes, schools, and food supplies were included and cross-verified with reports from Ukrainian ministries and local media.

Individual-level data on sleep and mental health (i.e., anxiety, depression, post-traumatic stress disorder, PTSD, and sleep quality) were collected through an online survey conducted from April 5 to May 15, 2023. An online quota sampling approach was used to collect data from 2,364 adult participants living in Ukraine (ages 18–79)^11,12^. Short sleep duration was defined as ≤ 6 hours, long as ≥ 9 hours, and insomnia was assessed using the Insomnia Symptom Questionnaire^12^. Winter infection data were also collected through another online survey from one adult (ages 18–72) per household in 2311 households across 24 Ukrainian oblasts. The questionnaire gathered information on respondents’ living and health conditions. Data on attacks on health facilities were extracted from the WHO databases^13^.

Ukrainian oblasts were categorized by region: North (Chernihiv, Sumy, and Kyiv), South (Odesa, Mykolayiv, Kherson, and Zaporizhzhia), East (Donetsk, Luhansk, and Kharkiv), and West (Zakarpattia, Chernivtsi, Lviv, and Volyn). We integrated survey-based health data, environmental indices, infrastructure accessibility, and casualty records across 461 Ukrainian cities. All data were cleaned, preprocessed, and structured for spatial analysis. The data categories and their corresponding explanatory variables are detailed in Table S1.

To estimate city-level prevalence, we applied three spatial interpolation methods: Inverse Distance Weighting (IDW), Empirical Bayesian Kriging (EBK), and Ordinary Least Squares (OLS). After comparing results, the IDW method was selected for final analysis. ArcGIS Pro’s Zonal Statistics tool was used to extract and estimate city-level prevalence values, including cold injury risk, based on IDW outputs. Key predictors in the model included insomnia scores^12^, sleep duration, environmental severity, housing vulnerabilities, and conflict intensity.

### Statistical Analysis

We calculated descriptive statistics, including mean, standard deviation, minimum, and maximum values for continuous variables, while percentages and total counts for categorical variables. Multivariable logistic regression was conducted to determine the association between mental health and sleep outcomes, housing and living conditions, infrastructure and essential services, and economic and social safety.

## Spatial Analysis

We used spatial regression to examine the relationships and spatial variability between mental health and sleep indicators as outcome variables. Explanatory variables included weapon types used in attacks, access to healthcare, medicine, food, humanitarian aid, heating equipment (e.g., logs/wood, coal, fuel briquettes, fuel pellets), ruined housing, and access to heat and water (see Table S1). Ordinary Least Squares (OLS) regression was used for spatial clustering, providing R^2^, adjusted R^2^, and p-values to assess model significance. All variables with a Variance Inflation Factor (VIF > 7.5) were dropped from models due to multicollinearity. OLS regression results are only reliable when the model is correctly specified^14^.

We also used a Geographically Weighted Regression (GWR) model. In geography, a strong predictor variable in one city may have little relevance in another city. GWR was used to explore this spatial heterogeneity, as it generates a unique regression equation for each geographic unit (i.e., city here). In this case, a city, weighting nearby observations more heavily in the calibration process. This approach allows model coefficients to vary across space, capturing localized relationships^15^.

## Vulnerability Index Creation

In addition, we combined the risk map with the sleep and mental health index to assess external stressors and internal responses. As described in more detail in the Supplement, this approach used principal component analysis (PCA) to integrate multiple dimensions to create a comprehensive vulnerability index (see supplement),^16^ including dependent (e.g., attack frequency) and independent variables (e.g., self-reported mental health) that capture the complex nature of vulnerability in conflict zones. A higher score or index indicates greater vulnerability.

## Suitability Modeling

Here, suitability modeling in GIS helps identify the areas most affected by war by examining the conditions and features of each place. We conducted suitability modeling using the tool in ArcGIS Pro. Composite indices were calculated for each variable class with PCA-derived weights in Python. Rasterized layers were combined using the weighted overlay tool to generate composite suitability maps for four distinct vulnerability categories.

### Model validation

The model was trained randomly on half of the cities selected for cross-validation and then tested on the remaining half. For bootstrapping, 100 random samples were drawn with replacement, and both models were applied to each sample. Across these iterations, over 95% of the explanatory variable coefficients remained statistically significant, demonstrating the models’ robustness and consistency.

### Ethical approval

The study protocol received ethical approval from the Poltava State Medical University Ethics Committee in Ukraine (Approval No. 212) and the Institutional Review Board at Rutgers University in the United States (Protocol #Pro2023000101).

## Results

A total of 1,934 drone attacks occurred from February 2022 to December 2023, resulting in 8,378 casualties (an average of 220.5) in 22 of 28 oblasts (Table S2A). Additionally, 1,162 attacks with other weapons that killed and injured people were also reported. A total of 258 artillery strikes were recorded, averaging 6.8 casualties per attack. PTSD (26.2%), depression (43.6%), anxiety (22.8%), loneliness (39.1%), and insomnia (36.7%) were reported (Table S2B).

### Regression analysis

People living in oblasts that experienced cold, damp, and crowded conditions were more likely to have mental health issues (Table S3). In multivariable models, individuals living in cold (Adjusted odds ratio, AOR 1.55, 95% CI: 1.16–2.06), damp (AOR 1.70, 95% CI: 1.29–2.24), and crowded (AOR 1.44, 95% CI: 1.10–1.87) conditions had significantly higher odds of PTSD. The odds of depression were similarly higher in those living in cold (AOR 1.57, 95% CI: 1.12–2.20), damp (AOR 1.80, 95% CI: 1.30–2.50), crowded (AOR 1.43, 95% CI: 1.05–1.93) conditions, needing house repairs (AOR 1.39, 95% CI: 1.00–1.93), and lacking housing subsidies (AOR 1.54, 95% CI: 1.11–2.12). Higher odds of anxiety, loneliness, and insomnia were found in cold, damp, crowded areas and those lacking housing subsidies. (Table S3).

People with less access to food, public transport, and who experienced frequent power outages had significantly higher odds of PTSD and loneliness (Table S4). However, lack of access to food shops and public transport was significantly associated with higher odds of depression, anxiety, and insomnia (Table S4).

Those with a lack of access to hospitals and ambulances were significantly associated with higher odds of PTSD, loneliness, insomnia, and depression, with anxiety linked only to a lack of access to ambulances (Table S5). Lastly, individuals who felt unsafe or insecure and lacked income had significantly higher odds of PTSD, while those feeling unsafe, insecure, and lacking food had significantly higher odds of depression, anxiety, loneliness, and insomnia (Table S6).

### Vulnerability Analysis

The analysis revealed significant spatial variability in mental health vulnerability in Ukraine. Severity, environmental conditions, and sleep and mental health indices were combined to create a comprehensive vulnerability map.

**Figure 1A.**
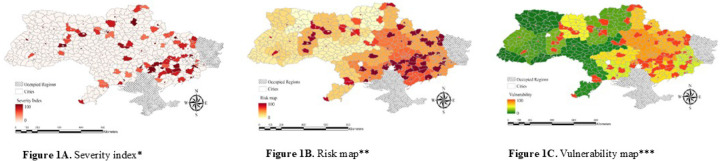
Severity Index, Figure 1B. Risk map, Figure 1C. Vulnerability map *Figure 1A: This index quantifies the intensity of war-related distuptions, including the frequency of attacks using artillery, shelling, aerial bombing, missiles, mining, multiple launch rocket systems, and drones, and the extent of casualties across cities. Regions with high values of this index correspond to areas that experienced the most severe conflict, with significant implications for mental health outcomes. **Figure 1B: Combines the severity (i.e., frequency of attacks using all weapon types and the extent of casualties) and environmental conditions indices (i.e., extreme cold, damp, frequent power outages, poor housing conditions, and crowded conditions), highlights regions at heightened risk due to a combination of conflict intensity and environmental exposure. ***Figure 1C: Integrates the risk map (all data used in Figure 1B) with the sleep and mental health index (i.e., anxiety, depression, and sleep deprivation), providing a comprehensive assessment of population vulnerability.

The Severity Index (Figure 1A) quantified war-related disruptions, including attack frequency and casualties, highlighting regions with severe conflict and mental health impacts. The Environmental Conditions Index (Figure S1) measured stressors like extreme cold, poor housing, and power outages, with high values in areas with severe winter and poor living conditions. The Sleep and Mental Health Index (Figure S2) reflected anxiety, depression, and sleep deprivation, with elevated scores indicating higher psychological distress. The Risk Map (Figure 1B) combined the Severity and Environmental Conditions indices, identifying regions at high risk due to conflict and environmental exposure. The Vulnerability Map (Figure 1C) integrated the Risk Map and Sleep and Mental Health Index, offering a comprehensive assessment of population vulnerability.

### Spatial analysis

The OLS model revealed key relationships between explanatory variables and mental health outcomes, with significant coefficients (Table S7). Given the model’s specifications, spatially adaptive modeling was required, leading to the application of GWR. GWR estimated regression coefficients for each spatial unit (i.e., city), capturing local vulnerability and mental health outcome variations. Model diagnostics indicated that spatially varying coefficients improved the model’s performance compared to the global OLS model (Table S8). The GWR analysis showed significant regional variation in vulnerability indices’ influence on mental health, highlighting the importance of location-specific factors during conflict.

**Figure 2.**
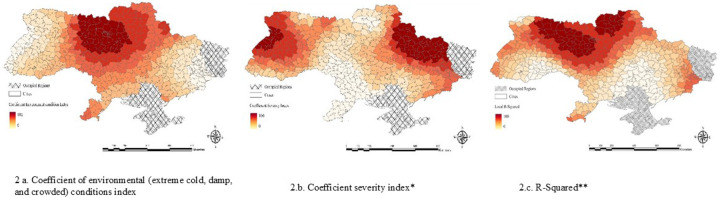
GWR Coefficients and R-Squared, 2.a. Coefficient Environmental Conditions Index, 2.b. Coefficient Severity Index, 2.c. R-Squared *The severity index represents conflict intensify (frequency of attacks, casualties, and destruction of houses). **The R^2^ map (Figure 2c) shows how well the model explains the variance in mental health outcomes across different regions.

The Environmental Conditions Index had the most substantial effect in the northern and central regions (Figure 2a), where harsh winters and poor infrastructure were associated with greater vulnerabilities. These areas showed higher rates of anxiety, depression, and sleep disturbances, with positive coefficients linked to poor mental health outcomes. The Severity Index, reflecting conflict intensity (attacks, casualties, and infrastructure damage), showed a substantial impact on mental health in eastern Ukraine (Figure 2b). Areas with prolonged violence and frequent attacks had higher risks of psychological distress, including PTSD and sleep deprivation, as indicated by high coefficient values. The Local R^2^ map (Figure 2c) showed the model’s explanatory power across regions. Central and eastern Ukraine had the highest values, indicating strong model fit, while western areas showed lower values, suggesting unmeasured factors influencing mental health. The variation in Local R^2^ suggests further investigation into factors affecting mental health in western Ukraine. This highlighted the spatial complexity of vulnerability and the need for tailored interventions.

A comparative analysis of the suitability maps showed significant overlap in the most vulnerable regions, particularly in eastern and southeastern Ukraine. These areas faced multidimensional risks from conflict, inadequate aid, environmental challenges, and housing damage, with the combined index offering a comprehensive assessment of mental health vulnerability.

### Vulnerability related to the availability of humanitarian assistance and financial support

**Figure 3.**
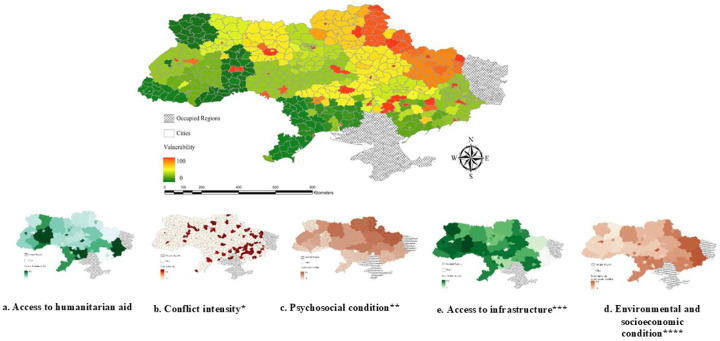
Vulnerability related to the availability of humanitarian assistance and financial support. 3.a. Access to Humanitarian Aid, 3.b. Conflict Intensity, 3.c. Psychosocial Condition, 3.d. Environmental and Socioeconomic Condition, 3.e. Access to Infrastructure Variables included *Frequency of attacks using artillery, shelling, aerial bombing, missiles, mining, multiple launch rocket systems, drones, and the casualties **PTSD, Depression, Anxiety, Loneliness, Insomnia, Sleep duration ***Public transport, hospital, pharmacy, ambulance, frequent outages of power ****Cold, damp, crowded, not safe and secure, not enough income/low income, not enough food, not enough medicines, not enough logs/wood, coal, fuel briquettes, fuel pellets, central heating and water supply system ruined by shelling, ruined house, need to insulate (repair) the house, no housing subsidy or its low level

Regions with limited access to humanitarian aid and financial support were more vulnerable (Figure 3). Key contributors to this vulnerability included the frequency of attacks and lack of access to essential services, such as food shops and public transport. Ukraine’s eastern and southeastern regions were the most affected, aligning with areas of high conflict intensity (Figure 3a–e).

### Mental Health vulnerabilities linked to environmental factors

**Figure 4.**
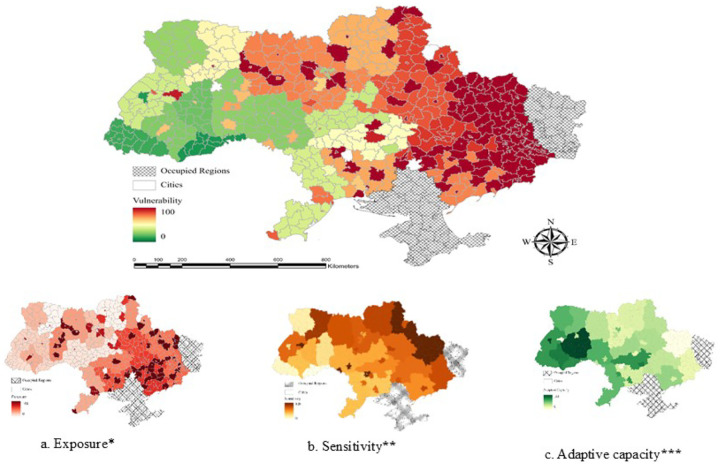
Health vulnerabilities linked to environmental factors. 4.a. Exposure, 4.b. Sensitivity, 4.c. Adaptive Capacity Variables included *Frequency of attacks using artillery, shelling, aerial bombing, missile, mining, MLRS, drone, causalities, cold, damp, crowded, not safe and secure, frequent outages of power, food shop, public transport, cash transfers or financial assistance, not enough food, not enough medicines, central heating and water supply system ruined by shelling, ruined house, need to insulate (repair) the house, no housing subsidy or its low level, and not enough logs/wood, coal, fuel briquettes, or fuel pellets **Not enough income/low income, PTSD, depression, anxiety, loneliness, insomnia, sleep duration ***Hospital, pharmacy, ambulance, and humanitarian aid

Environmental stressors like cold, damp housing, and frequent power outages significantly increased vulnerability in northern and eastern regions (Figure 4a–c). Sensitivity indicators, such as insufficient food and medicine, worsened health risks, while adaptive capacity indicators, like access to hospitals and pharmacies, were limited in highly vulnerable areas.

### Vulnerability related to housing damage

**Figure 5.**
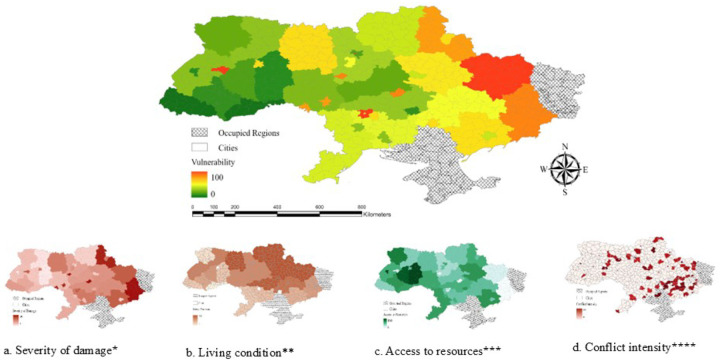
Vulnerability related to Housing damage. 5. a. Severity of Damage, 5.b. Living conditions, 5.c. Access to Resources, 5.d. Conflict Intensity Variables included * Ruined house, need to insulate (repair) the house, no housing subsidy or its low level ** PTSD, depression, anxiety, loneliness, insomnia, sleep duration, cold, damp, crowded, not safe and secure, not enough income/low income, not enough food, not enough medicines, central heating and water supply system ruined by shelling, and not enough logs/wood, coal, fuel briquettes, fuel pellets *** Frequent outages of power, food shop, public transport, hospital, pharmacy, ambulance, humanitarian aid, cash transfers, or financial assistance **** Frequency of attacks using artillery, shelling, aerial bombing, missiles, mining, multiple launch rocket systems, drones, and the casualties

The spatial distribution of housing vulnerability reflected the severity of housing damage and access (or lack thereof) to essential recovery resources (Figure 5). Housing vulnerability was highly correlated with conflict intensity, measured by attack frequency and weapon types. Regions with extensive housing damage and limited insulation repairs were particularly at risk during winter (Figure 5a–d).

### Health and food security vulnerability

**Figure 6.**
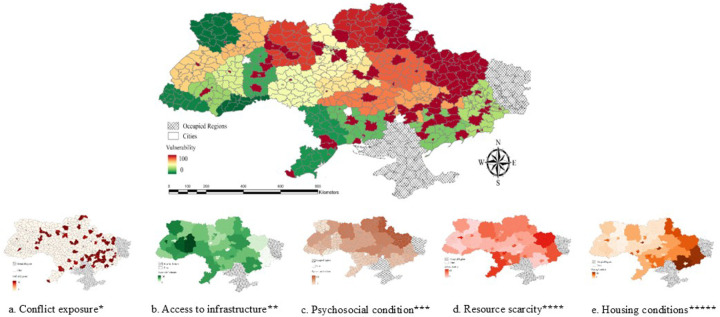
Health and Food Security Vulnerability. 6.a. Conflict Exposure, 6.b. Access to Infrastructure, 6.c. Psychosocial Condition, 6.d. Resource Scarcity, 6.e. Housing Conditions Variables included *Frequency of attacks using artillery, shelling, aerial bombing, missiles, mining, multiple launch rocket systems, drones, and the casualties **Frequent outages of power, food shop, public transport, hospital, pharmacy, ambulance, humanitarian aid, cash transfers or financial assistance, central heating, and water supply system ruined by shelling ***PTSD, depression, anxiety, loneliness, insomnia, sleep duration ****Not enough income/low income, not enough food, not enough medicines, and not enough logs/wood, coal, fuel briquettes, fuel pellets *****Cold, damp, crowded, not safe and secure, ruined house, need to insulate (repair) the house, no housing subsidy or its low level

Conflict exposure and food supply disruptions were key drivers of vulnerability, as shown in Figure 6, highlighting regions impacted by resource scarcity and health effects. Conflict exposure, access to food, and poor housing conditions worsened mental health outcomes, especially in areas with limited access to clean water, heating, and medical services (Figure 6a – e).

## Discussion

Using complementary data from the mainstream media, the Institute for the Study of War, UNICEF, and our cross-sectional data on winter infection, sleep, and mental health, we observed that environmental and conflict-related factors significantly affect sleep and mental health outcomes in Ukraine during the current conflict. Cold, damp, and overcrowded housing, along with limited access to heating, healthcare, food, and transport, were linked to higher rates of PTSD, depression, anxiety, loneliness, and insomnia, particularly in northern and rural regions. Conflict intensity exacerbated mental health issues in eastern Ukraine. Environmental stressors such as cold, damp, and frequent power outages appeared to be most impactful on mental health in northern Ukraine, while conflict severity was most important in the east.

As the first analysis in an active war zone, it provides insights into how psychological distress varies across different geographic and contextual factors in Ukraine’s ongoing conflict.

In 2021, the Uppsala Conflict Data Program recorded 54 state-based conflicts^17^. Research examines armed conflict’s economic, cultural, and humanitarian effects, including impacts on growth and human development. Similar to our findings, their research highlights elevated mortality, disability, and disease rates, as well as a high prevalence of mental disorders in conflict zones, with one in five adults affected living in a conflict zone^18,19^. Consistently, previous studies have shown that socio-demographic and geographic factors shape risks, while displacement, family separation, and service barriers compound psychosocial challenges^20–22^. Building upon that prior work, here we integrated spatial regression and suitability modeling to advance understanding of mental health and vulnerability in war-affected regions. This approach quantifies spatially distributed risk factors like proximity to risks and socioeconomic deprivation, providing deeper insights into localized mental health impacts.

Armed conflicts can devastate local environments, further exacerbating mental health issues in affected regions^23^. Eastern Ukraine, closer to the frontlines, is the target of frequent shelling, combat, and civilian-targeted violence^24^. Our results echo those findings, showing that eastern Ukraine endures direct conflict exposure, while also highlighting the spatial variation in vulnerability, such as northern Ukraine’s threats of environmental hazards, such as droughts and floods. This spatial granularity can inform humanitarian aid efforts, helping to prioritize regions with environmental and infrastructural vulnerabilities, not only in active conflict zones. Specifically, our results point to a need for improved heating and housing in northern Ukraine, while eastern regions may benefit most from enhanced PTSD treatment services. More generally, with limited resources in conflict-stricken areas, investment in mental health services may be informed by spatial analyses.

Prior conflict research suggests that high-intensity violence correlates with poor mental health outcomes, such as PTSD, depression, and anxiety^25^. However, other studies indicate that the relationship between conflict intensity and mental health is more complex. Some areas, despite high violence exposure, show better mental health outcomes^22^. Key factors include community resilience, community-based mental health interventions and access to mental health services, culturally grounded coping strategies, and humanitarian aid^26,27^. Strong local support systems can buffer the psychological impact of conflict, reducing the long-term mental health burden^28^. Additionally, areas with robust health infrastructure and services experience lower mental disorder prevalence, even in high-conflict regions^29,30^. Adequate physical and adaptive infrastructure resilience helps mitigate societal vulnerability and supports health^31^.

Compared with previous studies, our findings reveal that mental health burden is not directly tied to proximity to conflict. Northern oblasts, such as Chernihiv and Sumy, despite moderate attack intensity, experienced high anxiety, depression, and insomnia. Environmental stressors, including cold, housing damage, and poor access to essentials, significantly impacted mental health. Conversely, despite heavy shelling, some eastern areas had lower distress levels due to better humanitarian access. Our findings show that multiple vulnerabilities, such as housing destruction and supply disruptions, jointly predict increased risks of PTSD, depression, and insomnia. Spatial models also showed significant regional variations, underscoring the need for location-specific mental health and humanitarian interventions.

Previous studies confirmed that war-related environmental stressors significantly impact mental health and contribute to PTSD^32^. Our study also revealed that environmental conditions, such as extreme cold, poor housing, and power outages, may impact mental health more strongly than direct conflict exposure in some regions. Northern Ukraine, facing harsh winters and infrastructure failures, shows mental health vulnerabilities comparable to or exceeding areas with heavy shelling. In eastern Ukraine, a high-conflict area, PTSD prevalence reaches 26.2%, while northern Ukraine shows higher rates of depression (43.6%) and insomnia (36.7%). Factors like cold, dampness, and overcrowding contribute significantly to these outcomes. Indirect stressors, such as food insecurity and inadequate healthcare, also play a critical role.

Compared with the previous studies^11,12,33^ the GWR model in this study reveals significant spatial variation in mental health vulnerability across Ukraine. In eastern regions, PTSD and sleep deprivation correlate with conflict exposure, while in northern areas, anxiety and depression are linked to environmental factors. These findings emphasize the need for region-specific interventions.

A vulnerability index identifies high-risk communities for targeted aid and preparedness in war and public health crises^34^. Our study introduced a composite vulnerability index that integrates war severity, environmental stressors, and mental health indicators, providing a comprehensive risk assessment in conflict zones. It combines psychosocial, infrastructural, environmental, and conflict factors into a geospatial metric, highlighting compounded vulnerability in central and northern rural areas. The findings challenge traditional health frameworks by emphasizing localized conditions, such as infrastructure breakdown and other hardships (e.g., access to food shops and medicine), over direct conflict exposure, revealing spatial variation in mental health outcomes.

Wartime aid aims to save lives and uphold dignity, yet existing studies lack robust methods to support the best use and optimal distribution of humanitarian assistance during the conflict^35^. Our study reveals that aid distribution often overlooks areas that suffer from conflict and systemic hardships. These regions face severe shortages of essentials such as food, water, medicine, and heating, worsening psychological distress, PTSD, anxiety, and insomnia. Mental health may deteriorate further under conditions of uncertainty, neglect, and housing damage. Current aid models focus narrowly on combat zones and may miss broader geographic concerns, compounding vulnerabilities. Humanitarian efforts must prioritize regions with both physical insecurity and infrastructure collapse. Tailored interventions such as trauma therapy in conflict zones and housing support elsewhere are essential. Integrating multidimensional vulnerability indices can vastly improve aid allocation and promote health equity.

This study introduces a novel approach to modeling mental health vulnerability in conflict zones, combining geographically weighted regression, suitability mapping, and stressor indices.

This study has limitations, including recall bias in self-reported data and underrepresentation of isolated populations. The cross-sectional design doesn’t capture temporal fluctuations in vulnerability. This study used estimated city-level prevalence data. However, it carefully evaluated three interpolation techniques and chose the most widely used method that ensured precise, strong, and credible results compared with the two other approaches. The approaches here may be generalizable, but the specific pattern of results may be specific to the local context and this conflict in Ukraine.

## Conclusion

The findings highlight the need for adaptive frameworks in conflict-zone health research. A combined approach using spatial and suitability modeling offers a novel, multidimensional method for assessing vulnerability. Future research should explore the relationship between conflict intensity, environmental factors, and mental health to improve interventions. Applying this framework to other war-affected regions may enhance its utility. Mental health should be viewed not only as a trauma outcome but also as a reflection of systemic resilience and infrastructure stability. A shift in intervention strategies from a conflict-centric approach to a multi-dimensional resilience-building strategy that integrates environmental adaptation, mental health support, and infrastructure recovery is needed in Ukraine.

## Supplementary Material

Supplementary Files

This is a list of supplementary files associated with this preprint. Click to download.

- SupplementText.docx
- SupplementaryTables06.09.2025.docx
- SupplementFigures.pdf

## Data Availability

The data supporting this study’s findings will be available on request from the corresponding author.
